# Dopaminergic Neurotransmission in Patients With Parkinson's Disease and Impulse Control Disorders: A Systematic Review and Meta-Analysis of PET and SPECT Studies

**DOI:** 10.3389/fneur.2018.01018

**Published:** 2018-12-04

**Authors:** Alice Martini, Denise Dal Lago, Nicola M. J. Edelstyn, Matteo Salgarello, Fabio Lugoboni, Stefano Tamburin

**Affiliations:** ^1^School of Psychology, Keele University, Newcastle-under-Lyme, United Kingdom; ^2^Department of Nuclear Medicine, Ospedale Sacro Cuore Don Calabria, Verona, Italy; ^3^Addiction Unit, Department of Internal Medicine, University Hospital of Verona, Verona, Italy; ^4^Department of Neurosciences, Biomedicine and Movement Sciences, University of Verona, Verona, Italy

**Keywords:** Parkinson's disease, impulse control disorder, dopamine, PET, SPECT, transporter, receptors, meta-analysis

## Abstract

**Background:** Around 30% Parkinson's disease (PD) patients develop impulse control disorders (ICDs) to D_2/3_ dopamine agonists and, to a lesser extent, levodopa. We aim to investigate striatal dopaminergic function in PD patients with and without ICD.

**Methods:** PubMed, Science Direct, EBSCO, and ISI Web of Science databases were searched (from inception to March 7, 2018) to identify PET or SPECT studies reporting striatal dopaminergic function in PD patients with ICD (ICD+) compared to those without ICD (ICD–). Studies which included drug naïve patients, explored non-pharmacological procedures (e.g., deep brain stimulation), and those using brain blood perfusion or non-dopaminergic markers were excluded. Standardized mean difference (SDM) was used and random-effect models were applied. Separate meta-analyses were performed for dopamine transporter level, dopamine release, and dopamine receptors availability in the putamen, caudate, dorsal, and ventral striatum.

**Results:** A total of 238 studies were title and abstract screened, of which 19 full-texts were assessed. Nine studies (ICD+: *N* = 117; ICD–: *N* = 175 patients) were included in the analysis. ICD+ showed a significant reduction of dopamine transporter binding in the putamen (SDM = −0.46; 95% CI: −0.80, −0.11; *Z* = 2.61; *p* = 0.009), caudate (SDM = −0.38; 95% CI: −0.73, −0.04; *Z* = 2.18; *p* = 0.03) and dorsal striatum (SDM = −0.45; 95% CI: −0.77, −0.13; *Z* = 2.76; *p* = 0.006), and increased dopamine release to reward-related stimuli/gambling tasks in the ventral striatum (SDM = −1.04; 95% CI: −1.73, −0.35; *Z* = 2.95; *p* = 0.003). Dopamine receptors availability did not differ between groups. Heterogeneity was low for dopamine transporter in the dorsal striatum (*I*^2^ = 0%), putamen (*I*^2^ = 0%) and caudate (*I*^2^ = 0%), and pre-synaptic dopamine release in the dorsal (*I*^2^ = 0%) and ventral striatum (*I*^2^ = 0%); heterogeneity was high for dopamine transporter levels in the ventral striatum (*I*^2^ = 80%), and for dopamine receptors availability in the ventral (*I*^2^ = 89%) and dorsal (*I*^2^ = 86%) striatum, putamen (*I*^2^ = 93%), and caudate (*I*^2^ = 71%).

**Conclusions:** ICD+ patients show lower dopaminergic transporter levels in the dorsal striatum and increased dopamine release in the ventral striatum when engaged in reward-related stimuli/gambling tasks. This dopaminergic imbalance might represent a biological substrate for ICD in PD. Adequately powered longitudinal studies with drug naïve patients are needed to understand whether these changes may represent biomarkers of premorbid vulnerability to ICD.

## Introduction

Impulse control disorders (ICD), such as pathological gambling, hypersexuality, binge-eating, and compulsive shopping are diagnosed in around 30% of patients with Parkinson's disease (PD) (1–4).

ICDs are considered a complication of D_2/3_ dopamine agonist treatment and, to a lesser extent, levodopa (5). This is evident from studies showing higher ICD rates in medicated PD patients compared to healthy controls (2, 3, 6, 7). Although ICD rates have not been directly compared between medicated and drug naïve PD patients, other studies have shown that rates in drug naïve PD patients do not differ from healthy controls (8, 9). There are also retrospective case reports (10–12) and prospective studies (13–15) showing that in some cases ICDs onset (10–12, 14, 15) and their reduction or resolution (10, 13, 16) covary with dopaminergic treatment.

Preclinical animal studies provide further evidence of a modulatory effect of dopamine agonists on impulsivity using delay discounting paradigms. In these paradigms, impulsivity results in a behavioral preference for an immediate (smaller) reward over a delayed (larger) reward. However, the direction of the effect of dopamine on the reward system is inconsistent. For example, some studies showed lower levels of impulsivity on 1 and 2 mg/kg doses of *d*-amphetamine (17, 18) whereas others report increased impulsivity in rats treated with similar or higher doses (e.g., 0.8, 1, 1.20, 3.2 mg/kg) (19–21) or no effects (21, 22).

Studies in healthy volunteers show a modulatory effect of dopamine agonists on impulsivity; however, like rodent studies previously mentioned, the direction of the effect is unclear, in that some studies report increased impulsivity while other ones show decreased impulsivity to dopamine agonists. For example, *d*-amphetamine decreases impulsive behavior on the Stop task and in the Go/no Go task (measured as Stop reaction time and number of false alarms), and decreases delay discounting (23). However, other dopaminergic agents such as levodopa and pramipexole increase impulsivity on delay discounting and gambling tasks (24, 25).

In summary, evidence from preclinical rodent studies and healthy volunteers indicate that dopamine agonists modulate the reward system and impulsivity, but the direction of the effect is not clear. This implies that impulsivity is modulated by a complex interplay of dopamine activity across a network of systems, and dopamine agonists disrupt the balance between brain areas modulating impulsivity.

In the first stages of PD, the function of ventral striatum is relatively more preserved than the dorsal striatum (26). Therefore, the dopaminergic treatment dose required to restore motor dorsal striatal dopaminergic levels may overstimulate the relatively intact ventral striatum (27). This hyperdopaminergic state may promote an abnormal activity in the connected cortico-striatal cognitive and limbic pathways that mediate reward-related behavior (28). As a consequence, the control of goal-directed behavior is impaired, facilitating ICD development.

If ICDs in PD are linked to the disruption of the equilibrium in dopamine activity across ventral and dorsal striatum, then brain positron emission tomography (PET) and single-photon emission computed tomography (SPECT) can provide a direct measurement of putative dopaminergic differences between PD with and without ICDs. These nuclear medicine techniques use molecular imaging to assess biochemical, neurochemical, or pharmacological processes in the brain. For example, changes in neurotransmission can be detected using radiotracers with high affinity for dopamine receptors.

When a radiotracer is injected, it competes with dopamine for binding to free dopamine receptors. Thus, if dopamine is released endogenously, radiotracer binding can therefore be used as a marker for dopamine release (29). According to the binding affinity and the type of radiotracer, it is possible to investigate the nature of the dopaminergic dysfunction, whether linked to dopamine release, dopaminergic re-uptake in the presynaptic terminals, and D_2/3_ post-synaptic receptors availability. The spatial resolution of current PET and SPECT machines allow separate assessment of the dorsal and ventral striatal regions, and their components (i.e., putamen, caudate).

A limitation of the PET and SPECT studies of ICD in PD published so far is the small sample size, with the largest study including 21 PD patients with ICD and 68 without ICD (30) and the smallest including 7 PD patients with ICD and 7 without ICD (31). Small sample sizes are not surprising, given the high cost of PET and SPECT exams. Moreover, variability in clinical and demographic characteristics, types of tracer, protocols of analysis, and scanners makes the comparison between studies difficult.

A meta-analytic approach can overcome these limitations. Low powered studies can be combined and differences in striatal dopaminergic function between PD patients with and without ICD estimated with a higher reliability. To the best of our knowledge, no previous meta-analysis has been published on this topic.

Therefore, the objective of this study was to investigate differences in dopaminergic function in the striatum in PD patients with and without ICD. To this aim, we systematically reviewed and meta-analyzed PET and SPECT based reports on dopamine transporter level, presynaptic dopamine release, and post-synaptic D_2/3_ receptors availability in the ventral and dorsal striatum.

## Materials and Methods

### Search Strategy

The PubMed, Science Direct, EBSCO, and ISI Web of Science databases were searched for peer-reviewed studies on PET or SPECT striatal dopaminergic function in PD-related ICD and published from database inception until the 7th of March 2018.

**Figure 1:**
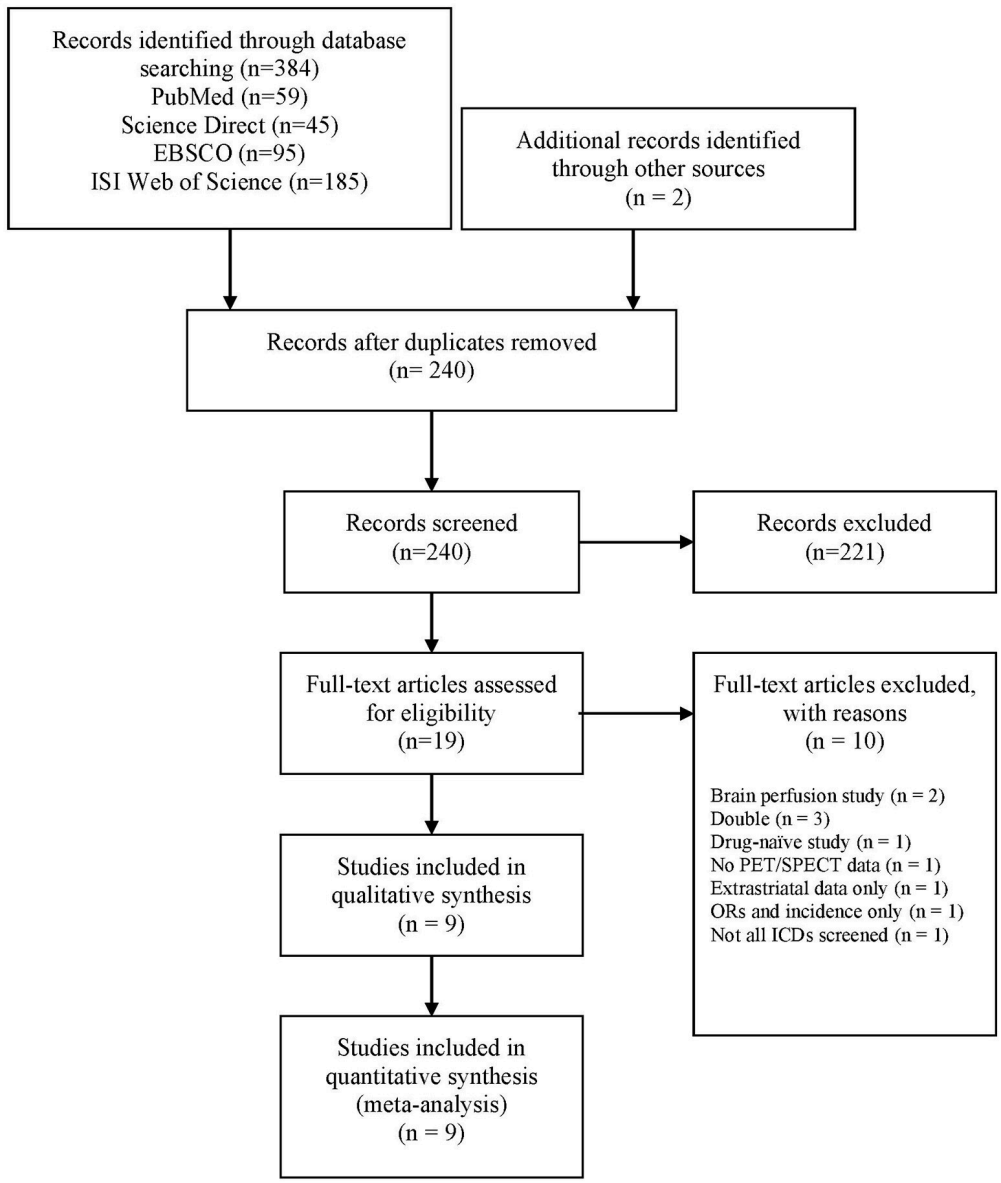
PRISMA diagram of the study (www.prisma-statement.org). ICD, impulse control disorder; ORs, odds ratios; PET, positron emission tomography; SPECT, single-photon emission computed tomography.

The following search string was used: “[(Parkinson's disease OR Parkinson) AND (impulse control disorders OR impulse control disorder OR impulsive compulsive behaviors OR impulsive compulsive behaviors OR impulsive compulsive behavior OR impulsive compulsive behavior OR ICD OR ICB OR hypersexuality OR gambling OR buying OR shopping OR eating)] AND (Positron emission tomography OR PET OR Single Photon Emission Computed Tomography OR SPECT OR SPET OR DaTSCAN).” A total of 384 papers were identified. After the exclusion of duplicates, 238 papers went through title and abstract screening. Two authors (AM, DDL) independently screened titles and abstracts using Rayyan software (32) and 17 papers were included in the full-text screening. The reference lists of these papers were manually searched for additional studies missed in the databases search, and two relevant papers were included at this stage. Two authors (AM, DDL) independently evaluated the 19 papers selected for full-text examination and disagreements were planned to be resolved via discussion with a third author (ST). However, there was 100% agreement between the two authors. Nine studies were included for quantitative analysis (Figure 1).

### Selection Criteria

Studies were included if they met the following inclusion criteria: (i) PET or SPECT study; (ii) PD patients without ICD (ICD–) compared with PD patients with ICD (ICD+); (iii) data reported for at least one striatal region; (iv) independence of the sample. Therefore, if a study sample was reported in multiple publications, only the study with the largest sample was included.

We excluded reviews, case studies, commentaries, letters, abstracts and dissertations, conference papers, and postal surveys. Studies including drug naïve PD patients were excluded, as we were interested on ICD developed after dopamine replacement treatment (DRT) initiation. Moreover, drug naïve PD patients represent a different sample than those treated with DRT, as the former have shorter PD duration, and are not chronically exposed to DRT. Therefore, dopaminergic systems may be affected and stimulated differently in medicated and non-medicated PD patients.

Studies in which PD patients underwent deep brain stimulation (DBS) were also excluded, as ICDs may either improve or develop after DBS (33). Finally, studies using measures of brain blood perfusion were excluded, as they do not explore striatal dopaminergic functioning. Similarly, we excluded studies with non-dopaminergic markers.

### Data Extraction

Corresponding authors of four studies (30, 34–36) were contacted for exact data. Data reported as median and range (37) were converted to mean and SD, as proposed by Hozo et al. (38). When standard error was reported, it was converted to SD (31, 39). Two authors (AM, DDL) independently extracted the following demographic and clinical data: sample size, sex, age at evaluation, age at PD onset, PD duration, education (years), Hoehn and Yahr (H&Y) stage, Unified PD Rating Scale motor section (UPDRS-III) ON-medication, depression, antidepressant use, antipsychotic use, number of patients under dopamine agonist treatment, dopamine agonist levodopa equivalent daily dose (LEDD, mg), levodopa LEDD, total LEDD, ICD screening tool, and ICD type. Methodological characteristics of the included studies were also extracted: imaging technique (i.e., PET or SPECT), type of tracer, reference region, imaging approach, radiotracer delivery method, drug delivered prior to scan, outcome measure, and striatal division and subdivision that was examined (i.e., ventral striatum, dorsal striatum, putamen, caudate).

The outcomes measures were the differences in the dopaminergic imaging parameters (e.g., binding potentials) between PD patients with and without ICD in striatal areas (i.e., ventral striatum, dorsal striatum, putamen, caudate).

### Data Analysis

Separate meta-analyses were performed for studies focusing on dopamine transporter level, dopamine release (presynaptic), and dopamine receptors availability (postsynaptic) in the ventral striatum, dorsal striatum, putamen, and caudate. Data were analyzed using ReviewManager v5.3 (40). Standardized mean difference (SMD) was used as effect size measure, with values around 0.2, 0.5, and 0.8 considered as small, moderate, and large, respectively (41). Heterogeneity between studies was calculated by the *I*^2^ value with percentages around 25, 50, and 75 considered as low, moderate, and high, respectively (42). As PD samples may vary in their clinical (e.g., H&Y stage, UPDRS scores) and demographic characteristics (e.g., age, sex), a random-effect model was applied.

Sensitivity analysis was performed by excluding studies clearly stating current antipsychotic or antidepressant use, as these drugs may affect dopamine receptor binding potential (43) or DAT uptake (44). As the number of studies was low, we lacked the power for conducting moderator analysis (45), or visual inspections of funnel plots for publication bias (46). A *p* < 0.05 was used as statistical significance threshold for all the analyses.

## Results

Demographic, clinical and methodological characteristics of the 117 ICD+ and 175 ICD– PD patients reported in the nine studies included in the meta-analysis are reported in Tables 1, 2.

**Table 1:** Demographic and clinical characteristics of the studies included in the meta-analysis.

**References**	**Pts (males)**	Age (y)*	PD onset (y)*	PD duration (y)*	Education (y)*	H&Y*	UPDRS-III (ON)*	**Depression as exclusion criteria?**	**Antidepressant use (N)**
Cilia et al. (34)	ICD+: 8 (7) ICD–: 21 (11)	ICD+: 60.7 (7.5) ICD–: 60.1 (9.4)	NR	ICD+: 6.25 (2) ICD–: 6.1 (2.4)	NR	ICD+: 2.1 (0.74) ICD–: 2 (0.53)	ICD+: 18.1 (9.3) ICD–: 20.2 (5.6)	NO (GDS available)	NO
Joutsa et al. (37)	ICD+: 10 ICD–: 10	ICD+: 61.5 (45-7)^¶^ ICD–: 61.5 (53-7)^¶^	ICD+: 53 (40-64)^¶^ ICD–: 57 (47-63)^¶^	ICD+: 7 (3-9)^¶^ ICD–: 5 (1-8)^¶^	NR	All patients were in stages 2 to 3	ICD+: 31 (24-41)^¶^ ICD–: 32 (19-49)^¶^	NO	NR
Lee et al. (56)	ICD+: 11 (8) ICD–: 11 (6)	ICD+: 56.6 (8.7) ICD–: 58.5 (7.3)	ICD+: 46.4 (8.7) ICD–: 49.2 (7.3)	ICD+: 10.1 (6.9) ICD–: 9.4 (2.3)	NR	ICD+: 2.3 (0.4) ICD–: 2.1 (0.5)	ICD+: 14.2 (11.0) ICD–: 15.3 (7.6)	YES	NO
Payer et al. (35)	ICD+: 11(9) ICD–: 21 (11)	ICD+: 58.9 (7.8) ICD–: 63.3 (8.7)	NR	ICD+: 12.1 (3.7) ICD–: 7.4 (4.5)	ICD+: 15.5 (2.7) ICD–: 15.1 (1.8)	NR	ICD+: 33.1 (10.2) ICD–: 28.1 (10.6)	YES	YES: ICD+: 1 ICD–: 0
Premi et al. (30)	ICD+: 21 (16) ICD–: 63 (38)	ICD+: 65.8 (8.4) ICD–: 68.5 (11.0)	NR	ICD+: 1.9 (2.2) ICD–: 1.7 (2.4)	NR	ICD+: stage 1 (*N* = 4); stage 1.5 (*N* = 3); stage 2 (*N* = 5); stage 2.5 (*N* = 5); stage 3 (*N* = 4) ICD–: stage 1 (*N* = 8); stage 1.5 (*N* = 13); stage 2 (*N* = 18); stage 2.5 (*N* = 17); stage 3 (*N* = 7)	ICD+: 16.5 (7.2) ICD–: 14.6 (7.7)	NO	YES: ICD+: 2 ICD–: 10
Stark et al. (36)	ICD+: 17 (11) ICD–: 18 (13)	ICD+: 60.9 (6.6) ICD–: 62.7 (10.1)	NR	ICD+: 5.7 (3.2) ICD–: 6.1 (4.5)	NR	NR	NR	YES	NR (unlikely considering the exclusion criteria)
Steeves et al. (31)	ICD+: 7 (5) ICD–: 7 (6)	ICD+: 47-72^§^ ICD–: 51-74^§^	NR	ICD+: 7.4 (3.2) ICD–: 5.6 (2.5)	NR	ICD+: 2 (0.6) ICD–: 1.9 (0.7)	OFF medication: ICD+: 25.2 (4.5) ICD–: 20.2 (5.4)	NO	NR
Voon et al. (44)	ICD+: 15 (9) ICD–: 15 (9)	ICD+: 55.1 (8.9) ICD–: 60.1 (8)	NR	ICD+: 7.5 (5.4) ICD–: 5.5 (5.2)	NR	ICD+: 3^†^ ICD–: 3^†^	NR	NR	NR
Wu et al. (39)	ICD+: 17 ICD–: 9	S-ICD: 62.3 (3.9)^||^ M-ICD: 58.1 (2.8)^||^ ICD–: 60.2 (3.2)^||^	S-ICD: 51.7 (4)^||^ M-ICD: 43.8 (3.4)^||^ ICD–: 50.3 (3.4)^||^	S-ICD: 10.6 (2)^||^ M-ICD: 14.3 (11.2)^||^ ICD–: 9.9 (2.1)^||^	NR	NR	OFF medication: S-ICD: 42.1 (3.8)^||^ M-ICD: 41 (3.5)^||^ ICD–: 32.8 (3)^||^	NO (but BDI scores available)	NR
**References**	**Antipsychotic use (N)**	**Drugs that may affect PET binding exclusion criterion**	**Patients under DA treatment (N/Total)**	**LEDD (mg)**			**Dementia excluded**	**ICD**	
				Total LEDD*	LD-LEDD*	DA-LEDD*		Diagnosis**	**Type: N**
Cilia et al. (34)	NO	YES	NR	ICD+: 831.2(293.6) ICD–: 852.3 (301.1)	NR	ICD+: 240.6 (118) ICD–: 251.6 (121)	YES (MMSE < 24)	Clinical interview (DSM-IV-TR criteria); SOGS	2 PG; 5 PG+HS; 3 PG+BE; 2 PG+CS
Joutsa et al. (37)	NR	NO but none used nicotine or had current substance-use disorder	ICD+: 9/10 ICD–:9/10	ICD+: 635 (250–876)^¶^ ICD–: 826 (210-1127)^¶^	NR	ICD+: 171.5 (0–280)^¶^ ICD–: 200 (0-320)^¶^	NO	Structured Clinical Interview for DSM-IV Axis I Disorders	4 PG;1 PG+subclinical HS; 1 HS; 2 HS+subclinical BE; 1 HS+subclinical CS; 1 BE
Lee et al. (48)	NO	YES	NR	ICD+: 914.4 (338.7) ICD–: 925.2 (458.4)	NR	ICD+: 217.9 (175.3) ICD–: 153.2 (110.7)	YES (MMSE < 24)	Clinical interview (DSM-IV-TR criteria); modified MIDI	1 HS; 2 PG; 3 CS+BE; 1 CS+HS+BE; 1 CS+HS+BE+PG; 2 CS+BE+punding; 1 HS+BE+PG
Payer et al. (35)	NR (unlikely considering the exclusion criteria)	YES. Current treatment with DA was exclusionary as it may interfere with ligand binding.	ICD+: 0/11 ICD–: 0/21	NR	ICD+: 813.6 (318.5) ICD–: 426.2 (144.6)	NR	YES (MMSE < 26)	clinical interview according to proposed criteria; SOGS; DSM-IV-based gambling questionnaire; sexual addiction screening test	10 PG; 3 HS; 1 CS. Only 5 patients meeting ICD criteria at the time of the study
Premi et al. (30)	NR	Antidepressant therapy, if present, was suspended 3 weeks before the assessment	ICD+: 19/21 ICD–: 30/63	ICD+: 594.2 (388.6) ICD–: 359.1 (280.1)	NR	ICD+: 282.1 (227.9) ICD–: 174.4 (97.2)	NO but MMSE scores reported	QUIP-rs	12 BE; 7 PG; 6 HS; 2 punding; 33 DDS + other ICDs; 1 DDS
Stark et al. (36)	NR (unlikely considering the exclusion criteria)	Patients excluded if they were prescribed psychoactive drugs that could alter dopamine receptor availability	ICD+: 17/17 ICD–: 18/18	ICD+: 673.8 (440) ICD–: 693.9 (406.3)	NR	ICD+: 103.9 (65.1) ICD–: 135.4 (76.4)	YES (MoCA < 22)	Clinical interview (DSM-IV-TR criteria); QUIP-rs;	11 HS; 11 BE; 4 CS; 12 hobbyism
Steeves et al. (31)	NR	NR	ICD+: 7/7 ICD–: 7/7	ICD+: 856 (407) ICD–: 756 (400)	NR	ICD+: 138 (172) ICD–: 167 (113)	YES	G-SAS	7 PG
Voon et al. (44)	NR	Patients were required to stop any drug that would bind to the DAT seven days prior to the scan	ICD+: 13/15 ICD–: 10/15	ICD+: 785.8 (402.7) ICD–: 852.1 (520.4)	NR	ICD+: 325.8 (156.1) ICD–: 384.3 (212.8)	NR	clinical interview	4 HS; 5 CS; 3 PG; 6 punding
Wu et al. (39)	NR	NR	NR	S-ICD: 782.3 (83.4)^||^ M-ICD: 724 (99)^||^ ICD–: 831.9 (119.2)^||^	S-ICD: 538 (83.4)^||^ M-ICD: 268.5 (84.9)^||^ ICD–: 666.3 (129)^||^	S-ICD: 244.3 (51.4)^||^ M-ICD: 244 (55.4)^||^ ICD–: 165.6 (48.8)^||^	YES (MMSE < 24)	semi-structured interview	4 HS; 3 PG; 1 CS+three ICDs; 2 CS+two ICDs; 7 CS +one ICD;

BDI, Beck depression inventory; BE, binge eating; CS, compulsive shopping; DA, dopamine agonist; DAT, dopamine transporter; DDS, Dopamine dysregulation syndrome; DSM-IV, diagnostic and statistical manual of mental disorders, fourth edition; DSM-IV-TR, diagnostic and statistical manual of mental disorders, fourth edition, text revision; G-SAS, Gambling symptom assessment scale; GDS, Geriatric depression scale; H&Y, Hoehn & Yahr score; HS, hypersexuality; ICD, impulse control disorder; ICD+, PD patients with ICD; ICD–, PD patients without ICD; LEDD, levodopa equivalent daily dosage (mg); LD, levodopa; M-ICD, multiple ICD; MIDI, Minnesota impulsive disorder interview; MMSE, mini mental state examination; MoCA, Montreal cognitive assessment; N, number of patients; NR, not reported. PD, Parkinson's disease; PET, positron emission tomography; PG, pathological gambling; Pts, patients; QUIP-rs, questionnaire for impulsive-compulsive disorders in Parkinson's disease rating scale; Ref, reference number; S-ICD, single ICD; SOGS, South Oaks gambling screen; UPDRS-III, unified Parkinson's disease rating scale part III (motor subscale) score; y: years. *Mean (SD) unless otherwise stated. ^¶^Median (range). ^§^Range. ^†^Median. ^||^Mean (SEM). **Questionnaire or method use to screen and/or diagnose ICD.

**Table 2:** Methodological characteristics of the studies included in the meta-analysis.

**References**	**Imaging technique**	**Type of tracer**	**Reference region**	**Imaging approach**	**Radiotracer delivery method**	**Drug delivered prior scan**	**ON/OFF**	**Withdrawal period**
Cilia et al. (34)	SPECT	[123I]FP-CIT	Occipital cortex	Single scan	Intravenous injection	Thyroid blockade (oral Lugol solution 10–15 mg) 30-40 min before the injection	OFF	Overnight withdrawal of dopaminergic medications
Joutsa et al. (37)	PET	[18F]fluorodopa	Occipital cortex	Single scan	Bolus injection	Carbidopa 150 mg 1h before the scan	OFF	At least 12 h drug discontinuation (>24 h for slow-release medications)
Lee et al. (48)	PET	[18F]FP-CIT	Cerebellum	Single scan	Bolus injection	NO	OFF	At least 12 h withdrawal of all PD medications
Payer et al. (35)	PET	[11C]-(+)-PHNO	Cerebellum	Single scan	Bolus injection	NO	OFF	At least 8 h withdrawal of levodopa (current DA use was an exclusion criteria)
Premi et al. (30)	SPECT	[123I]FP-CIT	Occipital lobe	Single scan	Intravenous injection	KClO_4_ 800 mg 30 min before the injection	NR	NR
Stark et al. (36)	PET	[18F]fallypride	Cerebellum	Three emissions scans	Bolus injection	NO	OFF	Washout was at least 40 h for DA and 16 h for levodopa
Steeves et al. (31)	PET	[11C]raclopride	Cerebellum	Two scans in two separate days within 2 weeks, in randomized order: baseline; gambling task	Ten mCi injections	NO	OFF	12–18 h overnight withdrawal of PD medications
Voon et al. (44)	SPECT	[123I]FP-CIT	Occipital lobe	Single scan	Slow intravenous injection	Thyroid blockade (oral potassium iodate) 24h prior to the study	ON	NO
Wu et al. (39)	PET	[11C]raclopride	Cerebellum	Two scans in 2 separate weekdays mornings: neutral stimuli; reward-related stimuli	Bolus injection	NO	OFF	12 h withdrawal of PD medications

*DA, dopamine agonist; mCi, millicurie; PD, Parkinson's disease; PET, positron emission tomography; SPECT, single-photon emission computed tomography*.

There was heterogeneity on the procedure to assess ICD across studies. ICDs were diagnosed either with a clinical interview based on the Diagnostic and Statistical Manual of Mental Disorders fourth edition text revision (DSM-IV-TR) (34–37, 44, 48), the Diagnostic and Statistical Manual of Mental Disorders fifth edition (DSM 5) (39) criteria or with the Questionnaire for Impulsive-Compulsive Disorders in PD—Rating Scale (QUIP-rs) (30). In four studies, the clinical interview followed the South Oaks gambling screen (SOGS) (34, 35), Gambling Symptom Assessment Scale (G-SAS) (31), QUIP-rs (36), and Sexual Addiction Screening test (35). In the paper of Steeves et al. (31), no specific information was provided on criteria for diagnosing ICDs apart from the use of SOGS for pathological gambling.

All patients were under DRT. In seven studies there were no between-group differences in total or dopamine agonist LEDD (31, 34, 36, 37, 39, 44, 48). One study reported a higher number of patients under dopamine agonist, however total LEDD and dopamine agonist doses were comparable between ICD+ and ICD– groups (30). In one study ICD+ had higher levodopa LEDD than ICD– (35).

One study divided the ICD+ group in single and multiple ICD subgroups (39). As the comparison between single and multiple ICD was not relevant for our meta-analysis, means and SDs of the subgroups were merged by calculating the pooled means and SDs.

Six studies provided results in the left/right (30, 34, 37, 39, 44, 48) and/or anterior/posterior striatal sub-regions (37); data from these studies were merged by calculating the pooled means and SDs.

Seven studies provided means and SDs for putamen and caudate separately (30, 35–37, 39, 44, 48). For these studies, putamen and caudate measures were merged to generate a measure of the whole dorsal striatum, according to Howes et al. (43). To this aim, the means of the dopaminergic index in the putamen and caudate were weighed by their volumes to reflect the larger contribution of the putamen compared to the caudate, and averaged (43). Since none of the studies reported the putamen and caudate anatomical volumes, we used those used by Howes et al. (43) and derived from healthy adults (*n* = 34, mean age = 32.5 years, *SD* = 8.8 years; mean, SD mm^3^ volume: putamen = 8805, 994; caudate = 5562, 865). SD was calculated accounting for the dependency of measures, by assuming a between-measures correlation of *r* = 0.5 in the striatal sub-regions. To test whether the whole dorsal striatum measure might have concealed differences in its sub-regions, analyses were repeated considering the putamen and caudate separately.

According to the radiotracer and the imaging approach used, studies were categorized as investigating (i) dopamine transporter level (30, 34, 37, 44, 48), (ii) dopamine release (31, 39), and (iii) dopamine receptors availability (31, 35, 36, 39). Information about radiotracers used in the studies included in the meta-analysis is reported in Table 3.

**Table 3:** Radiotracers used in studies included in the meta-analysis.

**Type of tracer**	**Study**	**Function and characteristics**
[123I]FP-CIT	Cilia et al. (34); Premi et al. (30); Voon et al. (44)	SPECT radiotracer with high affinity for DAT (49) and serotonin transporter (50)
[18F]FP-CIT	Lee et al. (48)	SPECT radiotracer with high affinity for DAT with high signal-to-noise ratio and kinetics (51)
[18F]fluorodopa	Joutsa et al. (37)	PET radiotracer for both presynaptic dopamine metabolism (synthesis) (52) and striatal dopamine uptake
[11C]raclopride	Steeves et al. (31); Wu et al. (39)	PET selective D2/D3 antagonist sensitive to changes in endogenous dopamine levels; it can be used to assess both basal levels of receptor availability and changes in availability caused by alterations in striatal dopamine concentration (53)
[11C]-(+)-PHNO	Payer et al. (35)	PET ligand with high affinity and selectivity for D_3_ receptors (54)
[18F]fallypride	Stark et al. (36)	PET ligand with high affinity to D_2/3_ receptors in striatal and extrastriatal regions (55)

*DAT, dopamine transporter; PET, positron emission tomography; SPECT, single-photon emission computed tomography*.

In the dopamine transporter level subgroup, three studies (30, 34, 44) used [123I]FP-CIT, a SPECT radiotracer with high affinity for DAT and modest affinity for the serotonin transporter (47); one study (48) used the [18F]FP-CIT radiotracer, which has also cross-affinity to serotonin transporter but a better contrast than [123I]FP-CIT (56); and one study (37) used [18F]fluorodopa, which is a marker of both dopaminergic re-uptake and dopamine synthesis (57).

The pre-synaptic dopamine release subgroup included two studies (31, 39) using [11C]raclopride, which is a competitive D_2/3_ antagonist sensitive to changes in endogenous dopamine levels (53). Both studies (31, 39) used a two PET sessions design, with one baseline scan [i.e., control task (31), neutral cues visual exposure (39)] and one scan during the experimental condition [i.e., gambling task (31), reward cues visual exposure (39)]. The binding potential in baseline condition is a measure of basal level of receptor availability. Conversely, the change in binding potential between baseline and experimental conditions is an indirect measure of alteration in striatal dopamine concentration due to pre-synaptic dopaminergic release. A decrease in binding potential in comparison to baseline is associated with increase in dopamine, while an increase in binding potential in comparison to baseline is associated with a dopamine decrease (53). Therefore, for the pre-synaptic dopamine release studies (31, 39), the outcome was the percentage [11C]raclopride binding potential reduction when comparing the experimental and baseline conditions.

Finally, the post-synaptic dopamine receptors availability subgroup included one study (35) with [11C]-(+)-PHNO, a D_3_-preferring D_2/3_ receptor ligand, and one study (36) with [18F]fallypride, which is one of the high affinity D_2/3_ receptor ligands that allow quantification of both striatal and extrastriatal binding. Two studies (31, 39) with [11C]raclopride were also included in the post-synaptic dopamine receptors availability analysis; for these studies the outcome was the value reported for the baseline conditions.

A total of 292 subjects were included in the meta-analysis, 117 were PD patients with ICD (age range: 45–72 years; PD duration: 1.9–14.3 years; H&Y: 2–3; UPDRS-III score ON medication: 14.2–41) and 175 were PD patients without ICD (age: 51–74 years; PD duration: 1–9.9 years; H&Y stage: 1.9–3; UPDRS-III score ON medication: 14.6–49) (Table 1).

Four meta-analyses were performed for the dopamine transporter levels in the ventral striatum, dorsal striatum, putamen, and caudate. Two meta-analyses were performed for the pre-synaptic dopamine release in the ventral and dorsal striatum; the putamen and caudate were not explored for this outcome, because only one study provided separate values for these two structures (39). Four meta-analyses were performed for the post-synaptic dopamine receptors availability in the ventral striatum, dorsal striatum, putamen, and caudate. Results of the meta-analyses are provided in Table 4.

**Table 4:** Results of the meta-analysis.

**Outcome**	**K**	***N***	**Random-effect model results**				**Heterogeneity**		
			**SMD**	**[95% CI]**	**Z**	***p***	*****X**^2^***	***p***	***I^2^*(%)**
Dopamine transporter level–ventral striatum	3	71	−0.91	[−2.10, 0.27]	1.51	0.13	10.14	*0.006*	80
Dopamine transporter level–dorsal striatum	5	184	−0.45	[−0.77, −0.13]	2.76	*0.006*	1.99	0.74	0
Dopamine transporter level–putamen	4	155	−0.46	[−0.80, −0.11]	2.61	*0.009*	1.43	0.70	0
Dopamine transporter level–caudate	4	155	−0.38	[−0.73, −0.04]	2.18	*0.03*	1.79	0.62	0
Dopamine release–ventral striatum	2	40	−1.04	[−1.73, −0.35]	2.95	*0.003*	0.22	0.64	0
Dopamine release–dorsal striatum	2	40	−0.36	[−1.01, 0.28]	1.10	0.27	0.42	0.52	0
Receptors availability–ventral striatum	4	107	−1.29	[−2.68, 0.10]	1.82	0.07	26.71	*< 0.00001*	89
Receptors availability–dorsal striatum	4	107	−0.69	[−1.86, 0.48]	1.16	0.25	21.90	*< 0.00001*	86
Receptors availability–putamen	3	93	−1.06	[−2.94, 0.81]	1.11	0.26	28.99	*< 0.00001*	93
Receptors availability–caudate	3	93	−0.59	[−1.40, 0.23]	1.41	0.16	6.86	*0.03*	71

*K, number of studies; N, number of participants; SMD, standardized mean difference; CI, confidence interval. P values below the significance level (p < 0.05) are reported in italics*.

### Dopamine Transporter Levels

**Figure 2:**
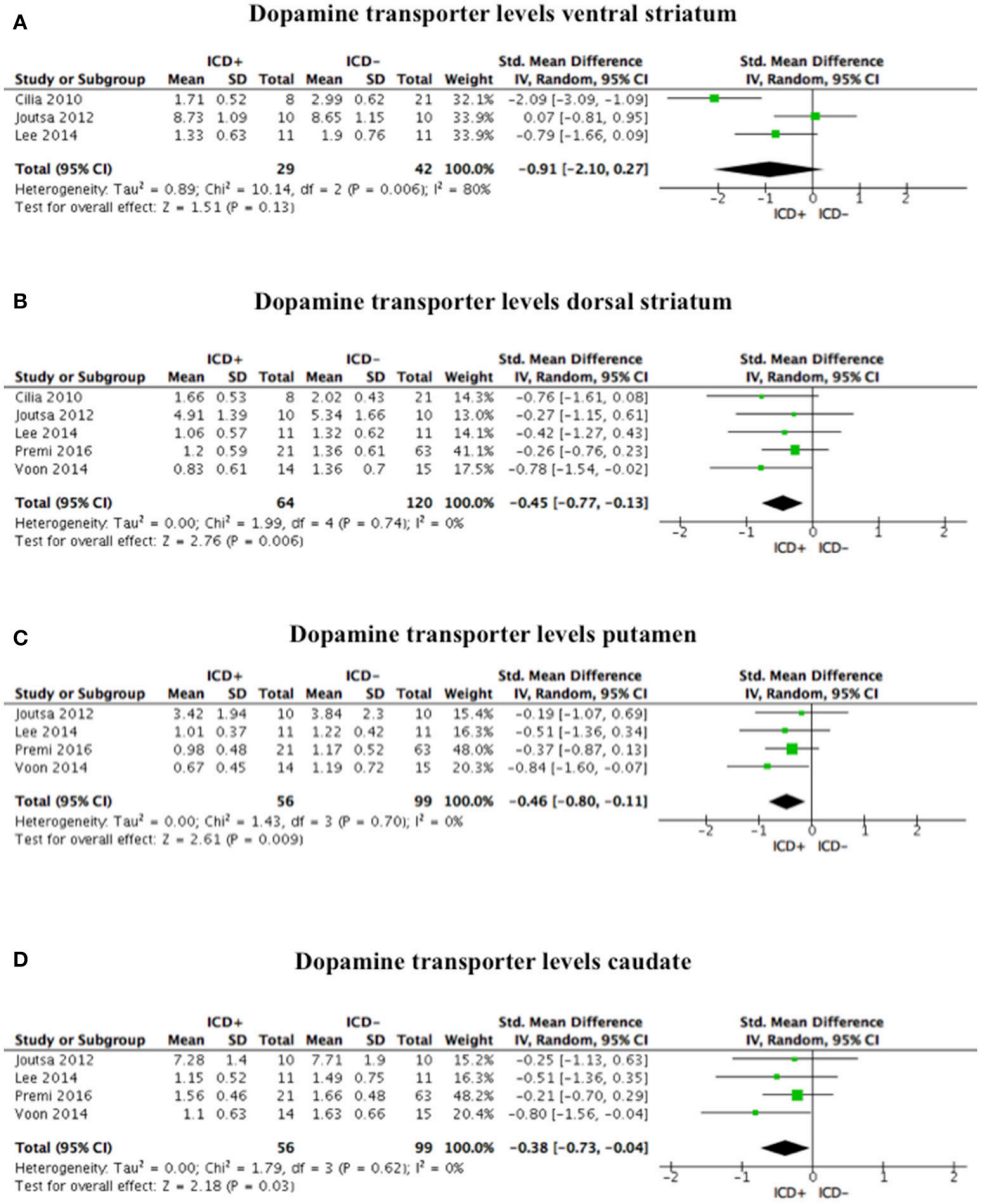
Forest plots for dopamine transporter levels. Here are reported forest plots for dopamine transporter levels in the ventral striatum **(A)**, dorsal striatum **(B)**, putamen **(C)**, and caudate **(D)**. Standardized mean difference represents Hedges's g effect size. The size of the square indicates the weight of the study. The horizontal line represents the 95% confidence interval. The diamond represents the pooled effect size. Negative effect sizes indicate lower dopamine transporter levels in PD patients with ICD (ICD+) in comparison to those without ICD (ICD–). ICD, impulse control disorder; PD, Parkinson's disease.

Compared to the ICD– group, tracer binding in the ICD+ group was significantly reduced in the dorsal striatum (SDM = −0.45; 95% CI: −0.77, −0.13; *Z* = 2.76; *p* = 0.006) but not in the ventral striatum (SDM = −0.91; 95% CI: −2.10, 0.27; *Z* = 1.51; *p* = 0.13). When dorsal striatum sub-regions were analyzed separately, both putamen (SDM = −0.46; 95% CI: −0.80, −0.11; *Z* = 2.61; *p* = 0.009) and caudate (SDM = −0.38; 95% CI: −0.73, −0.04; *Z* = 2.18; *p* = 0.03) tracer bindings were significantly reduced in the ICD+ vs. ICD– (Figure 2, Table 4).

Heterogeneity was low for the dorsal striatum (χ^2^ = 1.99, *p* = 0.74, *I*^2^ = 0%), putamen (χ^2^ = 1.43, *p* = 0.70, *I*^2^ = 0%), and caudate (χ^2^ = 1.79, *p* = 0.62, *I*^2^ = 0%). However, heterogeneity in the ventral striatum was high (χ^2^ = 10.14, *p* = 0.006, *I*^2^ = 80%; Figure 2). Sensitivity analysis was performed by excluding Premi et al. (30), which enrolled 12 patients under anti-depressant treatment that was suspended 3 weeks before assessment. Exclusion of Premi et al. (30) did not change overall effect size for dorsal striatum (SDM = −0.58; 95% CI: −0.99, −0.16; *Z* = 2.73; *p* = 0.006), putamen (SDM = −0.54; 95% CI: −1.02, −0.06; *Z* = 2.23; *p* = 0.03), and caudate (SDM = −0.54; 95% CI: −1.02, −0.07; *Z* = 2.24; *p* = 0.03), and heterogeneity (dorsal striatum: χ^2^ = 1.07, *p* = 0.78, *I*^2^ = 0%; putamen: χ^2^ = 1.19, *p* = 0.55, *I*^2^ = 0%; caudate: χ^2^ = 0.87, *p* = 0.65, *I*^2^ = 0%).

### Pre-synaptic Dopamine Release

**Figure 3:**
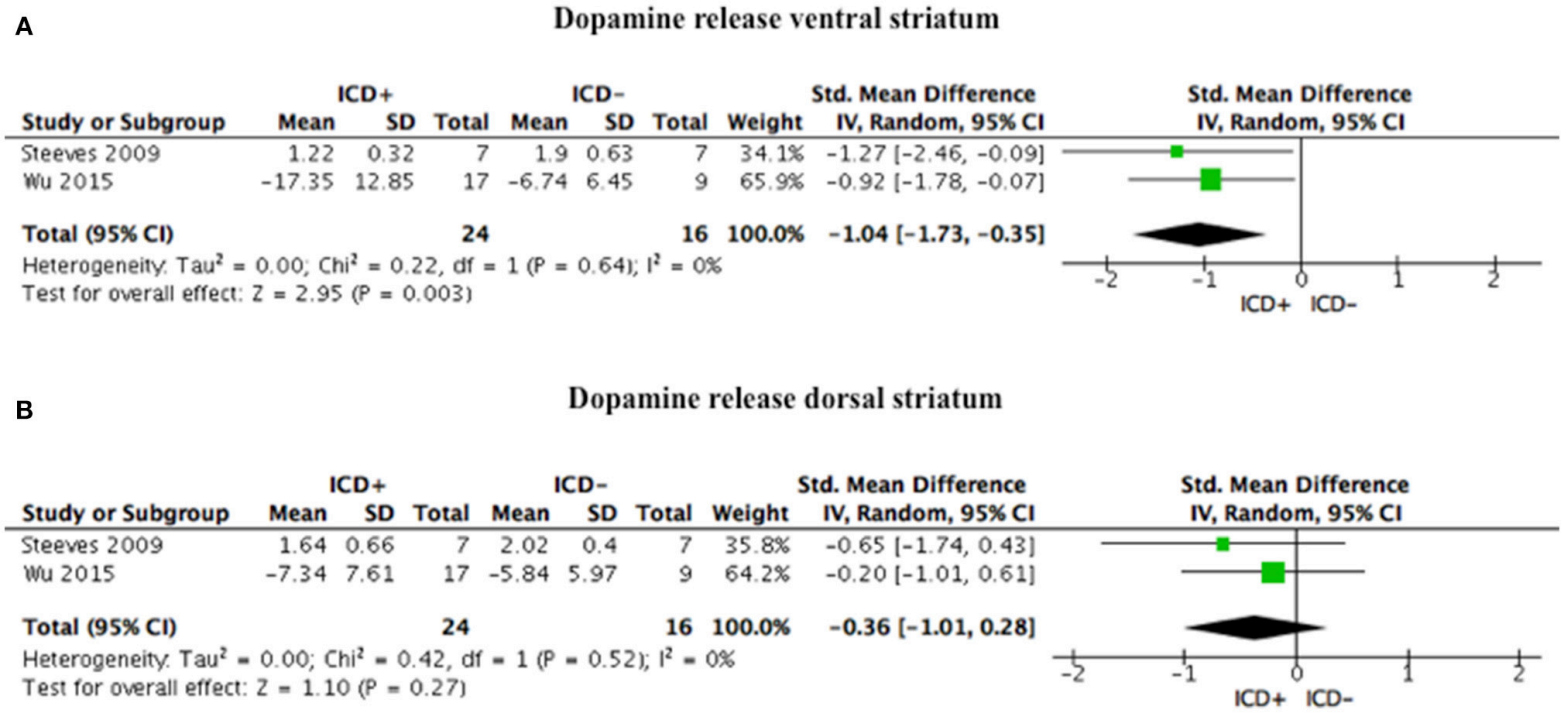
Forest plots for dopamine release. Here are reported forest plots for dopamine release in the ventral striatum **(A)**, and in dorsal striatum **(B)**. Standardized mean difference represents Hedges's g effect size. The size of the square indicates the weight of the study. The horizontal line represents the 95% confidence interval. The diamond represents the pooled effect size. Negative effect sizes indicate lower dopamine release in PD patients with ICD (ICD+) in comparison to those without ICD (ICD–). ICD, impulse control disorder; PD, Parkinson's disease.

ICD+ group, compared to the ICD– group, showed reduced binding in the ventral striatum in response to reward-related stimuli/gambling task (SDM = −1.04; 95% CI: −1.73, −0.35; *Z* = 2.95; *p* = 0.003), but not in the dorsal striatum (SDM = −0.36; 95% CI: −1.01, 0.28; *Z* = 1.10; *p* = 0.27; Figure 3, Table 4).

Heterogeneity was low for both ventral (χ^2^ = 0.22, *p* = 0.64, *I*^2^ = 0%) and dorsal (χ^2^ = 0.42, *p* = 0.52, *I*^2^ = 0%) striatal regions (Figure 3).

### Post-synaptic Dopamine Receptors Availability

**Figure 4:**
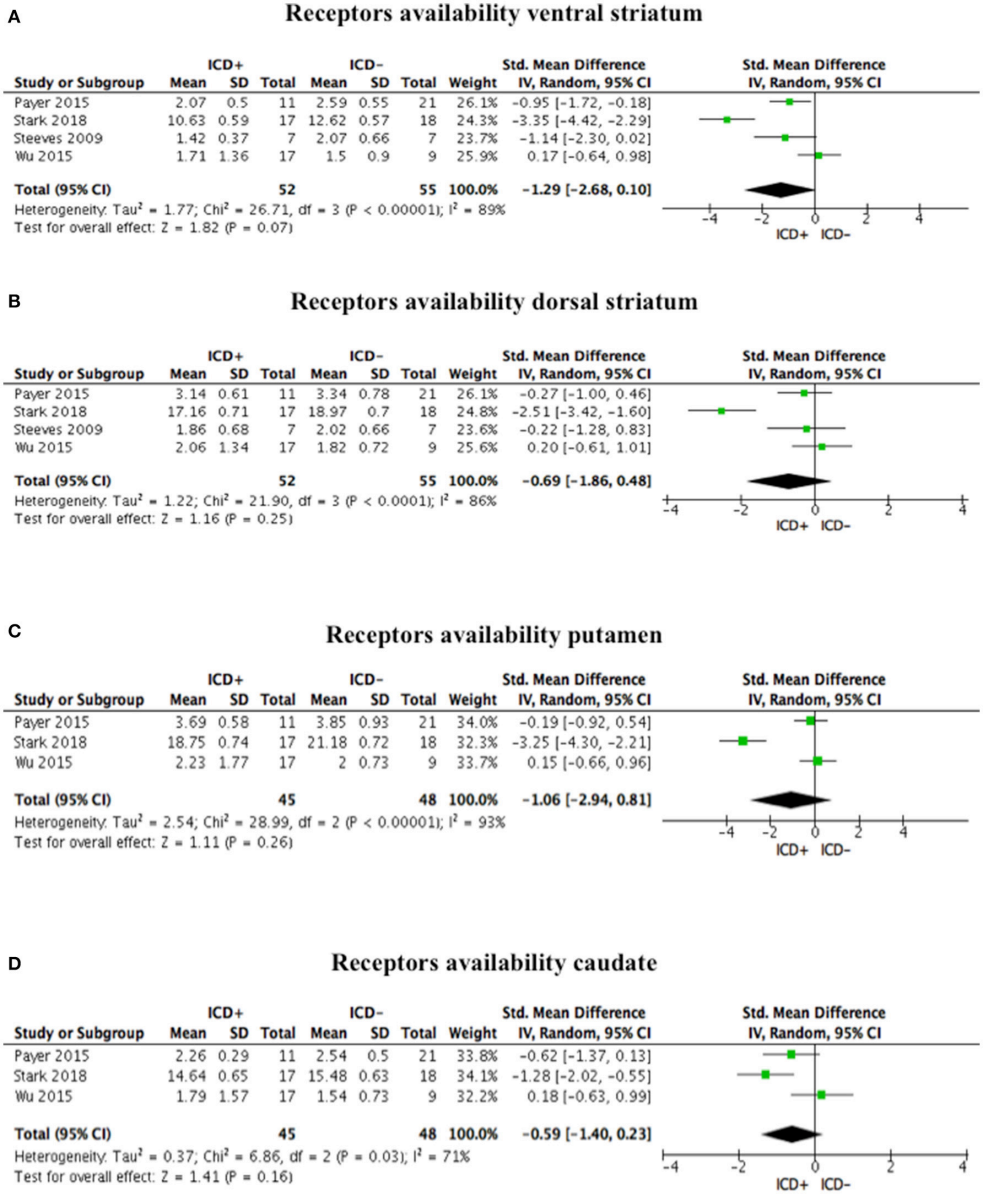
Forest plots for post-synaptic receptors availability. Here are reported forest plots for post-synaptic receptors availability in the ventral striatum **(A)**, dorsal striatum **(B)**, putamen **(C)**, and caudate **(D)**. Standardized mean difference represents Hedges's g effect size. The size of the square indicates the weight of the study. The horizontal line represents the 95% confidence interval. The diamond represents the pooled effect size. Negative effect sizes indicate lower receptors availability in PD patients with ICD (ICD+) in comparison to those without ICD (ICD–). ICD, impulse control disorder; PD, Parkinson's disease.

Post-synaptic dopamine receptor bindings potentials did not differ between ICD+ and ICD– groups in the ventral striatum (SDM = −1.29; 95% CI: −2.68, 0.10; *Z* = 1.82; *p* = 0.07), dorsal striatum (SDM = −0.69; 95% CI: −1.86, 0.48; *Z* = 1.16; *p* = 0.25), putamen (SDM = −1.06; 95% CI: −2.94, 0.81; *Z* = 1.11; *p* = 0.26), and caudate (SDM = −0.59; 95% CI: −1.40, 0.23; *Z* = 1.41; *p* = 0.16; Figure 4, Table 4).

Heterogeneity was high in the ventral striatum (χ^2^ = 26.71, *p* < 0.00001, *I*^2^ = 89%), dorsal striatum (χ^2^ = 21.90, *p* < 0.0001, *I*^2^ = 86%), putamen (χ^2^ = 28.99, *p* < 0.00001, *I*^2^ = 93%), and caudate (χ^2^ = 6.86, *p* = 0.03, *I*^2^ = 71%; Figure 4).

Sensitivity analysis was performed by excluding Payer et al. (35), which enrolled one patient taking antidepressant. Exclusion of Payer et al. (13) did not change the overall effect size for the ventral striatum (SDM = −1.42; 95% CI: −3.54, 0.69; *Z* = 1.32; *p* = 0.19), dorsal striatum (SDM = −0.84; 95% CI: −2.55, 0.86; *Z* = 0.97; *p* = 0.33), putamen (SDM = −1.54; 95% CI: −4.87, 1.80; *Z* = 0.90; *p* = 0.37), and caudate (SDM = −0.56; 95% CI: −1.99, 0.87; *Z* = 0.77; *p* = 0.44), and heterogeneity (ventral striatum: χ^2^ = 26.58, *p* < 0.00001, *I*^2^ = 92%; dorsal striatum: χ^2^ = 20.53, *p* < 0.0001, *I*^2^ = 90%; putamen: χ^2^ = 25.42, *p* < 0.00001, *I*^2^ = 96%; caudate: χ^2^ = 6.86, *p* = 0.009, *I*^2^ = 85%).

## Discussion

This is the first systematic review and meta-analysis on PET/SPECT dopaminergic striatal correlates of ICD in PD. Our aim was to investigate if striatal dopaminergic function differs in PD patients with and without ICD. To this aim, we reviewed and analyzed studies on dopamine transporter levels, presynaptic dopamine release, and post-synaptic D_2/3_ receptors availability in the ventral and dorsal striatum. We found ICD+ to be associated with (i) lower DAT levels in the dorsal striatum and in its subdivisions (i.e., putamen, caudate) and (ii) reduced binding (i.e., increased dopamine release) in the ventral striatum in response to reward-related stimuli or gambling task, but (iii) no relationship between ICD+ and striatal post-synaptic receptors availability in either the dorsal or ventral striatum.

### Dopamine Transporter Levels

ICD+ group showed lower dorsal striatum DAT binding than the ICD– one.

In the striatum, DAT is localized in axon varicosities and terminals that contain synaptic vesicles, as well as in non-synaptic region where it regulates and terminates extracellular dopamine activity (44). Therefore, reduced DAT might reflect more pronounced dorsal striatal dopaminergic terminal loss, functional DAT downregulation, or genetically determined lower membrane expression on otherwise normal neurons (34).

The hypothesis of more severe degeneration of nigrostriatal projections in ICD+ patients is supported by a recent meta-analysis of case-control studies showing that the risk of ICD in PD increases with disease duration and being medicated for PD (58), two factors that are directly correlated with the amount of nigrostriatal loss. Moderator analysis for these two factors was not possible, because of the small number of studies included in the meta-analysis.

The lower DAT binding in ICD+ may also reflect medication-related DAT downregulation, but DAT regulation by DRT was found to be modest (44, 59). It is unlikely that lower DAT binding is a compensatory effect of medication, as longitudinal studies on drug naïve PD patients show that dorsal striatal DAT downregulation precedes DRT initiation (15, 60). SPECT findings are further supported by a genetic study showing an association between ICD in PD and a variant of the dopamine transporter gene, i.e., 9-repeat allele of the SLC6A3 (61); this variant results in lower presynaptic DAT expression, reduced synaptic clearance, and increased DA availability in the synaptic space (62).

### Pre-synaptic Dopamine Release

ICD+ group showed reduced binding potential in ventral but not dorsal striatum when exposed to reward-related cues or when engaged in a gambling task.

Participants to a gambling task are required to actively choose options associated either with reward or penalty and process related feedback. Conversely, in reward-related cues paradigms, participants passively view neutral or reward-related stimuli (e.g., food, erotic pictures, gambling, or shopping related activities) without any active choice. Albeit being different, these tasks share neurobiological underpinnings. In pathological gamblers, reductions of ventral striatal and ventromedial prefrontal cortex activity have been documented in a gambling task (63) and reward-related reactivity has been shown to involve the dorsal lateral prefrontal cortex network (64) that is functionally connected to the ventral striatum.

[11C]raclopride is sensitive to competition from endogenously released dopamine to a stimulus, therefore decreased binding potential found in ICD+ vs. ICD– groups in response to gambling tasks or rewarding stimuli reflects increased dopamine release. These findings are in keeping with functional imaging studies of behavioral and pharmacological addiction in the general population, whereby monetary and sexual stimuli elicit the same patterns of striatal activation as recreational drugs (31, 65). Increased dopamine release during a gambling task has been reported in pathological gamblers (66, 67) and it correlates both with gambling severity (68) and increased excitement levels despite lower performances (66). This may be the consequence of conditioned response to the reward-related or gambling cues, although increased dopaminergic release has been observed also for unconditioned stimuli (39). Whether the increased dopamine release in the ventral striatum exists in the premorbid phase therefore representing a vulnerability factor or it is the consequence of repeated exposure to gambling or rewarding-related stimulus (69, 70), to DRT (71), or a combination of these factors (31) is unknown. Only preclinical models and prospective studies can address this point.

These findings have important implications, since the exposure to any reward-related cue (e.g., through advertisement) may have the potential to increase abnormal dopamine release in vulnerable PD patients (72), as supported by a study showing increased dopamine release in single ICD PD patients to reward-cues not related to their ICD (e.g., gamblers to food-related cues) (39).

There are two other neuropharmacological mechanisms that should be considered. First, in patients treated with dopamine agonists the activation of presynaptic D_2_-like presynaptic autoreceptors in the mesolimbic system reduces phasic dopamine release in the nucleus accumbens (25, 73, 74). Therefore, reward responsiveness is blunted and risk propensity enhanced in order to normalize mesolimbic efflux (73). Second, reward detection capacity depends on phasic dopaminergic cell firing. Phasic dopamine dips encode prediction errors therefore providing outcome-related feedback which signal the need of behavioral adjustments as reward contingencies change (75). In rats, a low dose of monoamine-depleting agent reserpine administered together with pramipexole, exacerbated its effects on disadvantageous decision-making without changing pramipexole-induced decrease in the phasic dopamine release. This suggests that the effect of dopamine agonist on ICD may not be caused by changes in phasic dopamine release in the nucleus accumbens (73). Moreover, dopamine agonists tonically bind to D_2_ receptors irrespective of phasic changes in firing (76).

### Post-synaptic Dopamine Receptors Availability

We did not find changes in D_2/3_ receptors availability between ICD+ and ICD– PD patients. This finding is, to some extent, surprising for a number of considerations. Animal PD models showed increased D_3_ expression after repeated administration of DRT (35). A PET study found relationships between higher D_3_ levels, dopamine release in the ventral striatum, and ICD severity in people without PD (77). Preclinical rats models of PD shows that ICD–like behaviors can be triggered by pramipexole (78, 79) and ropinirole (80, 81), which mainly target D_2/3_ receptors. Polymorphisms of D_2/3_ receptors genes are associated with addictive behaviors in PD (82), and in the general population (83). D_3_ receptor antagonists may block reward seeking in animal models (84–86).

Different lines of reasoning may explain this apparently paradoxical finding. Heterogeneity was high for this outcome in our meta-analysis, and this may reflect differences in the radiotracers used by the studies we included. However, random effect model does not assume homogeneity of the effect and findings should have been robust to heterogeneity. D_2/3_ receptors localize both to pre-synaptic mesolimbic terminal auto-receptors and post-synaptic indirect-pathway medium spiny neurons (36). Therefore, binding of radiotracers may reflect a mix of pre- and post-synaptic changes (35). Moreover, D_2/3_ receptors changes have not been universally observed across the spectrum of maladaptive reward-seeking behavior, where reductions are notably absent in primary gambling addiction (36, 87). In individuals with substance dependence there is lower D_2/3_ receptors availability than healthy controls (88), but no differences have been reported in pathological gamblers (66, 67).

### Limitations and Future Directions

The main limitation of the present meta-analysis is the small number of studies included, and consequently the low statistical power, which impede any definite conclusions on the mechanisms underlying ICD in PD. The small number of studies hampered a moderator analysis, which would have added information on the variables potentially contributing to our results. Our data suggest that more studies with large numbers of patients are needed. They should have a longitudinal design with drug naïve patients, to clarify the causative relations between striatal dopaminergic changes and ICD, and whether they are pre-morbid vulnerability traits, or a consequence of DRT. Current cross-sectional studies may only document associative links. Future studies should incorporate a healthy control group (34, 35, 48), as some dopaminergic changes might be age-related and not directly linked to PD or ICD (89).

PET/SPECT studies on extrastriatal regions, which interact with the striatum in the control of motivated and addictive behavior (37, 48), are still scarce, and focus on a range of different structures, impeding a meta-analysis. The role of extrastriatal dopaminergic changes should be assessed. At the time of our literature search, five studies reported data on extrastriatal regions, including the orbitofrontal (37), medial orbitofrontal (35), ventromedial prefrontal (48), and left anterior cingulate cortex (90), the amygdala (48), substantia nigra (35), globus pallidus (35, 36), ventral pallidus (35), thalamus (36), and the midbrain (36, 90). Exploring these areas would be important, since, e.g., abnormal functioning of D_2/3_ midbrain receptors might results in increased dopamine release (91).

Since the dopamine system may not be the only player in ICD development, multi-modal imaging studies should explore the contribution of serotoninergic systems to ICD in PD (30, 61, 92).

Finally, ICD in PD was found to be associated with cognitive (worse set-shifting and reward-related decision-making), and neuropsychiatric features (increased depression, anxiety, anhedonia, and impulsivity) (93). The potential confounding role of these clinical variables should be considered in future PET/SPECT studies.

## Conclusions

Our meta-analysis showed specific patterns of dopaminergic dysfunction in the dorsal and ventral striatum in PD patients with ICD. These changes, which, to some extent, differ from those in people with ICD but no PD, may reflect either a preexisting neural trait vulnerability for impulsivity or the expression of a maladaptive synaptic plasticity under non-physiological dopaminergic stimulation (30).

## Author Contributions

The study has been designed by AM, NE, and ST. Data have been gathered by AM and DDL, under the supervision of MS and ST. Data have been analyzed by AM. The manuscript has been drafted by AM, NE, and ST. AM, DDL, FL, MS, NE, and ST revised the manuscript.

### Conflict of Interest Statement

The authors declare that the research was conducted in the absence of any commercial or financial relationships that could be construed as a potential conflict of interest.
